# Dr. John O. Aldrich

**Published:** 1917-02

**Authors:** 


**Dr. John O. Aldrich,** Cincinnati 1883, died at his home in Bath, Steuben Co., N. Y., Jan. 18, aged 68.

